# Untargeted metabolomic profiling of urine from healthy dogs and dogs with chronic hepatic disease

**DOI:** 10.1371/journal.pone.0217797

**Published:** 2019-05-31

**Authors:** Yuri A. Lawrence, Blake C. Guard, Jörg M. Steiner, Jan S. Suchodolski, Jonathan A. Lidbury

**Affiliations:** Gastrointestinal Laboratory, Department of Small Animal Clinical Sciences, College of Veterinary Medicine and Biomedical Sciences, Texas A&M University, College Station, Texas, United States of America; University of Illinois, UNITED STATES

## Abstract

Chronic hepatic disease can present a diagnostic challenge with different etiologies being associated with similar clinical and laboratory findings. The histopathological assessment of a liver biopsy specimen is usually required in order to make a definitive diagnosis and the availability of non-invasive prognostic biomarkers is limited. The emerging science of metabolomics is used to detect changes in endogenous low molecular weight metabolites in biological samples and offers the possibility of identifying noninvasive markers of disease. The objective of this study was to investigate differences in the urine metabolome between healthy dogs, dogs with chronic hepatitis, dogs with hepatocellular carcinoma, and dogs with a congenital portosystemic shunt. Stored urine samples from 10 healthy dogs, 10 dogs with chronic hepatitis, 6 dogs with hepatocellular carcinoma, and 5 dogs with a congenital portosystemic shunt were analyzed. The urine metabolome was analyzed by gas chromatography–quadrupole time of flight mass spectrometry and 220 known metabolites were identified. Principal component analysis and heat dendrogram plots of the metabolomics data showed clustering between groups. Random forest analysis showed differences in the abundance of various metabolites including putrescine, gluconic acid, sorbitol, and valine. Based on univariate statistics, 37 metabolites were significantly different between groups. In, conclusion, the urine metabolome varies between healthy dogs, dogs with chronic hepatitis, dogs with hepatocellular carcinoma, and dogs with a congenital portosystemic shunt. Further targeted assessment of these metabolites is needed to assess their diagnostic utility.

## Introduction

Chronic hepatic disease in dogs includes chronic hepatitis: idiopathic chronic hepatitis, copper-associated chronic hepatitis, drug-associated chronic hepatitis; lobar dissecting chronic hepatitis, and granulomatous chronic hepatitis, breed-associated metabolic errors, congenital portosystemic vascular anomalies, and hepatocellular carcinoma [1,2]. Differentiating these diseases can pose a diagnostic challenge due to the similarities of clinical signs and laboratory findings between different diseases. Although an early accurate diagnosis is important for an improved clinical outcome, achieving a definitive diagnosis can be cost prohibitive and invasive with the histological examination of a liver biopsy specimen regarded as the gold standard. The identification of non-invasive biomarkers that can reliably characterize chronic hepatic disease is desirable and could have clinical implications.

The liver is a central organ for regulation of metabolism, and a number of metabolic disturbances occur in patients with chronic liver disease [3,4]. Data from studies in humans and animal models have documented alterations in hepatic lipid metabolism, protein metabolism, energy metabolism, cytokine metabolism, and increased generation of reactive oxygen species in patients with chronic liver disease [5–8]. Global metabolomic profiling, the untargeted quantification of small molecules in biologic samples, allows for a comprehensive analysis of changes in several metabolic and signaling pathways and their interactions [9–11]. This platform utilizes nuclear magnetic resonance spectroscopy or mass spectrometry to measure low molecular weight metabolites permitting the formation of a metabolite profile. Metabolite profiles can be altered by a variety of physiological and pathological processes, and therefore global changes in such profiles may signal the presence of a particular disease [12,13]. Characteristic metabolite profiles that can discriminate between various types of liver disease have been identified in human studies [14–19]. There are no previous studies that have evaluated the urine metabolome of dogs with chronic hepatic disease to the authors’ knowledge.

The aim of the present study was to compare the urine metabolome of healthy dogs, dogs with chronic hepatitis, dogs with hepatocellular carcinoma, and dogs with a congenital portosystemic shunt. We hypothesized that there would be a difference in urine metabolites between groups and that the four groups would have significantly different metabolomes.

## Materials and methods

### Patients and procedures

Adult dogs with definitively diagnosed chronic hepatic disease diagnosed at Texas A&M University Veterinary Medical Teaching Hospital or Gulf Coast Veterinary Specialists between January 2011 and December 2014 were enrolled in this prospective observational study. The diagnosis of chronic hepatitis was based on clinical signs, serum biochemical evaluation, diagnostic imaging findings, and the histological assessment of a liver biopsy specimen by a board-certified veterinary pathologist. Liver biopsy specimens were collected by diagnostic laparoscopy, where five to eight total specimens were collected from different liver lobes using forceps, or during celiotomy, where one to four wedge or punch biopsies were collected from one or more liver lobes. The liver biopsy specimens were fixed in neutral buffered formalin for routine histological processing. Histological sections from formalin-fixed paraffin embedded tissue were stained with hematoxylin and eosin. Additional stains were performed at the discretion of the anatomic pathologist and attending clinician. Dogs with a history of current systemic disease such as hyperadrenocorticism, cancer, or others were excluded from the study. Dogs with a suspected congenital portosystemic shunt were evaluated by ultrasonography as previously described [20]. Dogs that presented for a wellness examination to the Small Animal Hospital at Texas A&M University, College Station, Texas were used as healthy controls. The health of these dogs was assessed by performing a physical examination, owner questionnaire, complete blood count, and a serum biochemistry profile. Dogs with clinically important abnormalities were excluded from the study. The study was approved by the Texas A&M University Institutional Animal Care and Use Committee (Animal Use Protocol #2014–0320). Informed client consent was obtained for each dog prior to enrollment in the study.

### Medical record data collection

The information reported from the medical record of the healthy control dogs, dogs with chronic hepatitis, dogs with hepatocellular carcinoma, and dogs with congenital portosystemic shunting included breed, diet, and medical treatment at the time of enrollment.

### Urine sample collection

A urine sample was obtained from all dogs by cystocentesis. Urine samples were kept at ambient room temperature (approximately 22°C) for less than 30 minutes before being placed on ice, and then frozen within 1 hour of sampling for storage at -80°C. Samples collected at Gulf Coast Veterinary Specialists were kept at -80°C until shipment on dry ice to Texas A&M University, where they continued to be stored at -80°C. All dogs had free access to water; however, food was withheld for a minimum of 12 hours prior to urine sample collection.

### Urine metabolome analysis

Untargeted metabolomics analysis was performed by the West Coast Metabolomics Center at the University of California (Davis, CA) on a fee-for-service basis. Urine aliquots were normalized by urine creatinine concentration measured by a SIRRUS Clinical Chemistry Analyzer and extracted by degassed acetonitrile. Internal standards C8-C30 fatty acid methyl ethers were added, and the samples were derivatized by methoxyamine hydrochloride in pyridine and subsequently by N-methyl-N-trimethylsilyltrifluoroacetamide for trimethylsilylation of acidic protons. Analytes were separated using an Agilent 6890 gas chromatograph (Santa Clara, CA) and mass spectrometry was performed on a Leco Pegasus IV time of flight mass spectrometer (St. Joseph, MI) following a published protocol [21]. Unnamed peaks were excluded from statistical analysis.

### Statistical analysis

#### Univariate analysis

Univariate analysis was performed using JMP Pro 13 (JMP Software, SAS Software Inc., Marlow, England). The dataset was tested for normality using the Shapiro-Wilk test and because all metabolites did not meet the assumptions of normal distribution, comparisons between healthy dogs, dogs with chronic hepatitis, dogs with hepatocellular carcinoma, and dogs with a congenital portosystemic shunt groups were determined using the non-parametric Kruskal-Wallis test. The resulting p-values were adjusted for multiple comparisons using the Benjamini-Hochberg’s False Discovery Rate and significance was set at q < 0.10 [22]. Post hoc testing was performed with Dunn's Test of multiple comparisons using JMP Pro 13 and significance was set at q < 0.05.

#### Multivariate analysis

Data were reported as peak height. Principle component analysis, random forest analysis, and hierarchical cluster analysis was performed using MetaboAnalyst 4.0 (http://www.metaboanalyst.ca) after autoscaling as previously described [23,24].

## Results

### Selection criteria of case and control dogs

There were 10 healthy control dogs enrolled in the study based on a normal physical examination, owner questionnaire, complete blood count, serum biochemistry profile and the absence of any reported clinical signs. There were 10 dogs with chronic hepatitis and 6 dogs with hepatocellular carcinoma enrolled in the study based on histologic examination of a liver biopsy specimen. There were 5 dogs with a congenital portosystemic shunt enrolled in the study.

### Animal population

The demographics of the healthy control dogs, dogs with chronic hepatitis, dogs with hepatocellular carcinoma, and dogs with a congenital portosystemic shunt enrolled into the study are summarized in Table 1. Breeds of dogs included in each study group, diet, and medical treatment are summarized in Table 2. There was a significant difference in the distribution of age and sexes between groups.

**Table 1:** Summary of demographic information of dogs enrolled into the study.

Heading	Control (n = 10)	C. Hepatitis (n = 10)	H. Carcinoma (n = 6)	Congenital PSS (n = 5)	p-value
**Age in years (median, range)**	4.0, 1-8^A^	10.8, 2–12 ^AB^	10.5, 5-13^B^	3.0, 0.5–5 ^A^	0.001
**Sex (M/F)**	5/5^A^	5/5^A^	6/0^B^	5/0^B^	0.033

C. Hepatitis–Chronic hepatitis, H. Carcinoma–Hepatocellular carcinoma, Congenital PSS–Congenital portosystemic shunt, M–intact or castrated male, F–intact or spayed female, groups with same superscript are not significantly different.

**Table 2:** Healthy (n = 10), chronic hepatitis (n = 10), hepatocellular carcinoma (n = 6), congenital portosystemic shunt (n = 5) dog breeds, diet, and medical treatment at time of sample collection.

Group	Breed	Diet	Medical Therapy
**Healthy**	English Setter	Maintenance	None
**Healthy**	Golden Retriever	Maintenance	None
**Healthy**	Crossbreed Dog	Maintenance	None
**Healthy**	Crossbreed Dog	Maintenance	None
**Healthy**	Crossbreed Dog	Maintenance	None
**Healthy**	English Pointer	Maintenance	None
**Healthy**	Standard Schnauzer	Maintenance	None
**Healthy**	Crossbreed Dog	Maintenance	None
**Healthy**	Boston Terrier	Maintenance	None
**Healthy**	Boxer	Maintenance	None
**Chronic Hepatitis**	Crossbreed Dog	Maintenance	NC
**Chronic Hepatitis**	Bassett Hound	Maintenance	None
**Chronic Hepatitis**	Labrador Retriever	Hill’s l/d	None
**Chronic Hepatitis**	Cirneco Dell'Etna	Maintenance	NC, U
**Chronic Hepatitis**	Miniature Schnauzer	Maintenance	NC
**Chronic Hepatitis**	West Highland White Terrier	Maintenance	None
**Chronic Hepatitis**	Crossbreed Dog	Maintenance	NC
**Chronic Hepatitis**	Labrador Retriever	Maintenance	NC, U
**Chronic Hepatitis**	Yorkshire Terrier	Hill’s l/d	AB
**Chronic Hepatitis**	Australian Shepherd Dog	Maintenance	None
**Hepatocellular Carcinoma**	Crossbreed Dog	Maintenance	None
**Hepatocellular Carcinoma**	Chihuahua Dog	Maintenance	None
**Hepatocellular Carcinoma**	Crossbreed Dog	Maintenance	None
**Hepatocellular Carcinoma**	Siberian Husky Dog	Maintenance	None
**Hepatocellular Carcinoma**	Crossbreed Dog	Maintenance	AB
**Hepatocellular Carcinoma**	Chihuahua Dog	Maintenance	None
**Congenital Portosystemic Shunt**	Pug	Hill’s l/d	None
**Congenital Portosystemic Shunt**	Maltese Terrier	RC Hepatic	AB
**Congenital Portosystemic Shunt**	Rat Terrier	Hill’s l/d	None
**Congenital Portosystemic Shunt**	Miniature Schnauzer	RC Hepatic	AB, L
**Congenital Portosystemic Shunt**	Maltese Terrier	Maintenance	NC

AB–antibiotic therapy, L–lactulose therapy, NC–nutraceutical therapy (denamarin or s-adenosyl methionine), U–ursodiol, RC–Royal Canin.

### Differences in the urine metabolome between healthy control dogs, dogs with chronic hepatitis, hepatocellular carcinoma, and a congenital portosystemic shunt

**Fig 1:**
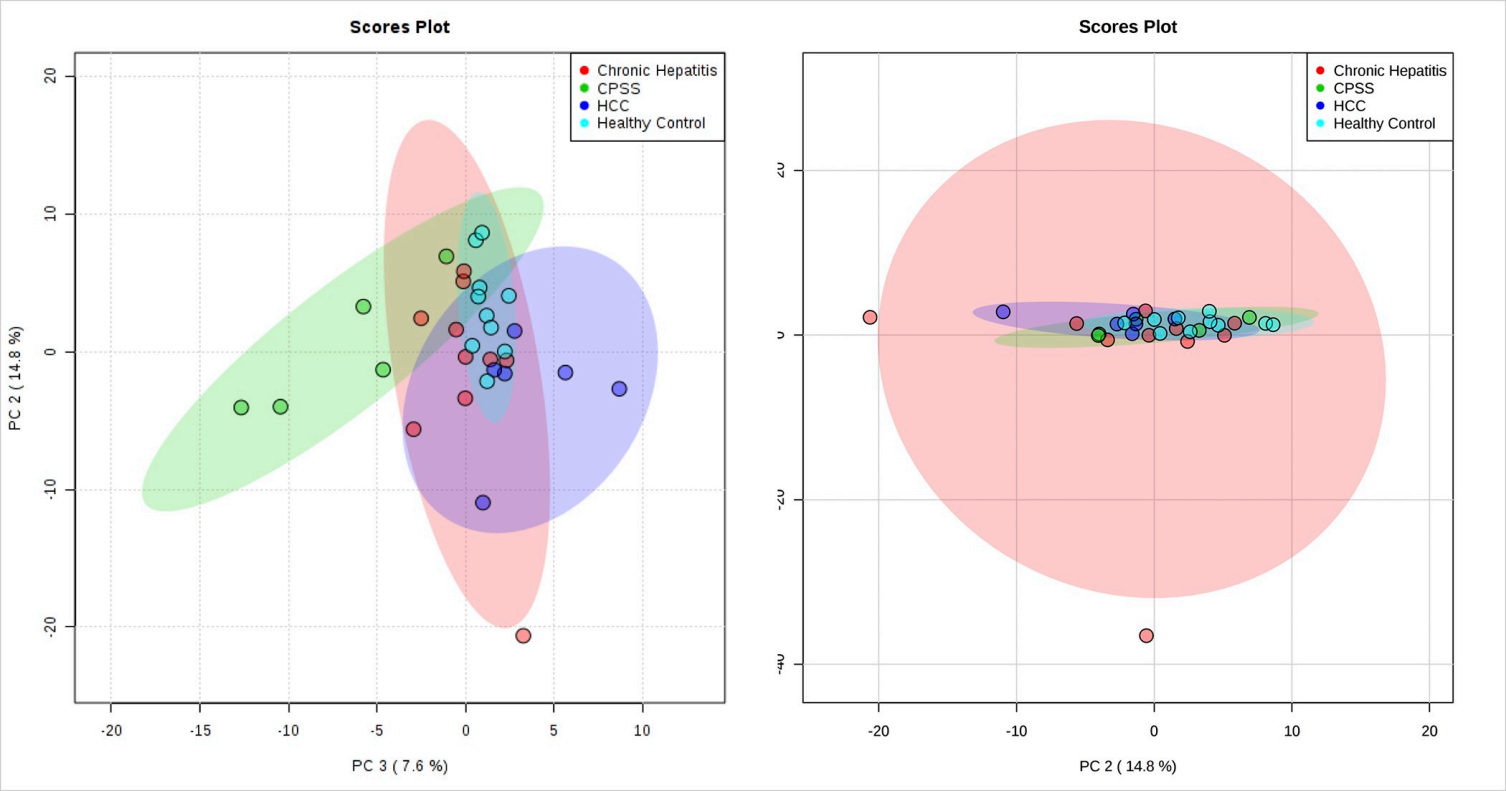
Principal component analysis of the urine metabolome showing clustering of samples based on group. The scores plot showed a clustering of groups. HCC–Hepatocellular carcinoma; CPSS–Congenital portosystemic shunt.

**Fig 2:**
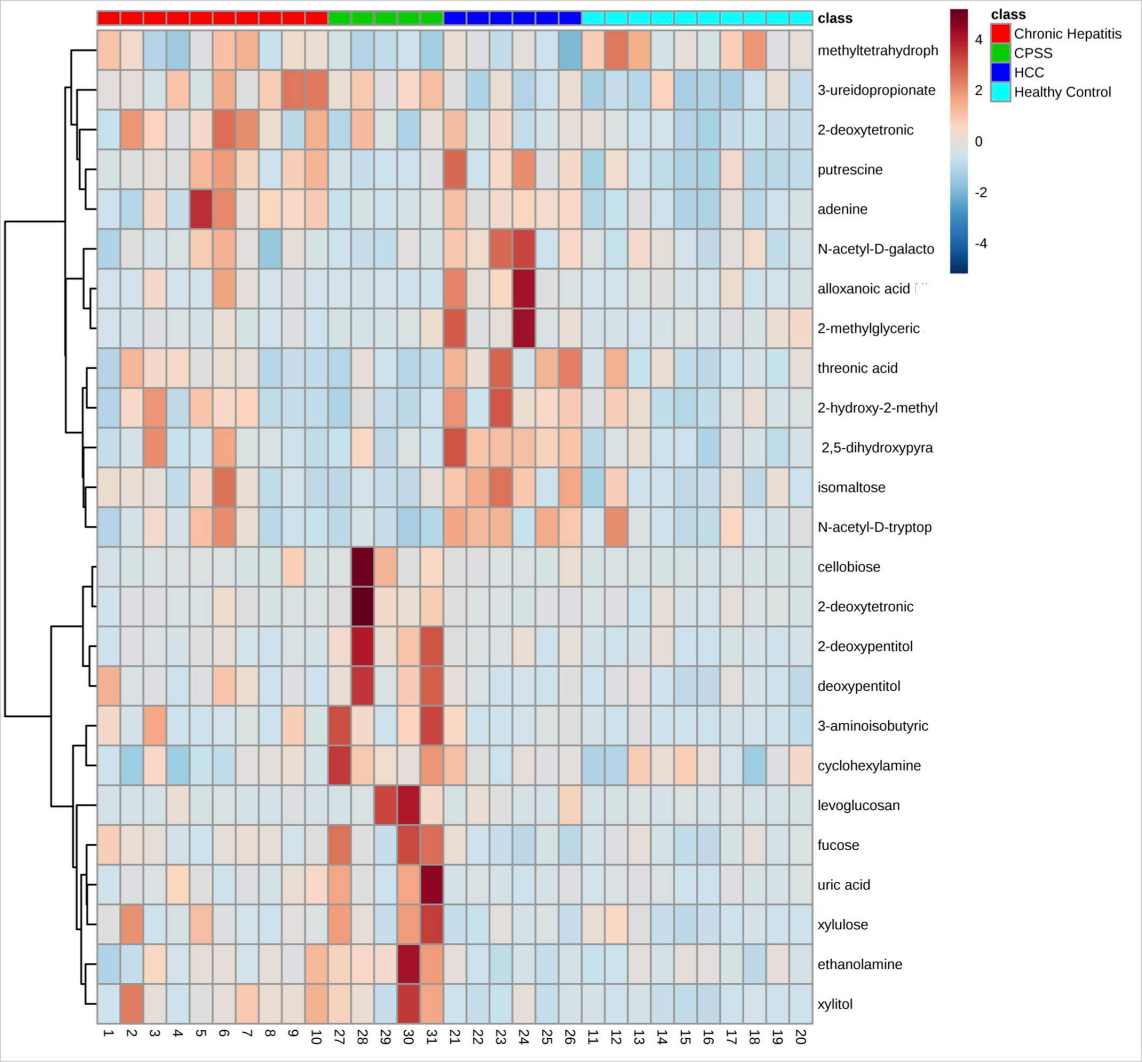
Heatmap of the most abundant metabolites in all groups, as identified by VIP scores in PLS-DA. Each sample is represented by a single column. The higher the intensity of the red color, the higher the abundance of the metabolite (HCC–Hepatocellular carcinoma; CPSS–Congenital portosystemic shunt).

**Fig 3:**
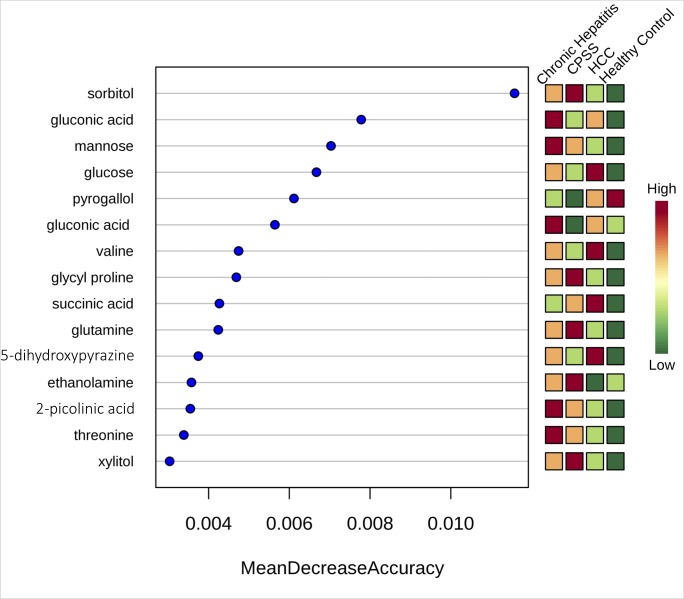
**Top 15 metabolites in urine (left panel) in rank order of importance for differentiating between healthy control dogs, dogs with chronic hepatitis, dogs with hepatocellular carcinoma (HCC), and dogs with a congenital portosystemic shunt (CPSS).** Data were generated by random forest analysis. Compounds contributing the most to the accuracy of disease classification were identified as those having the highest mean decrease accuracy (MDA) of the predicted classification when permuted within the dataset. These 15 metabolites were able to classify healthy control dogs (100% accuracy), dogs with chronic hepatitis (70% accuracy), dogs with hepatocellular carcinoma (33% accuracy), and dogs with a congenital portosystemic shunt (40% accuracy).

**Fig 4:**
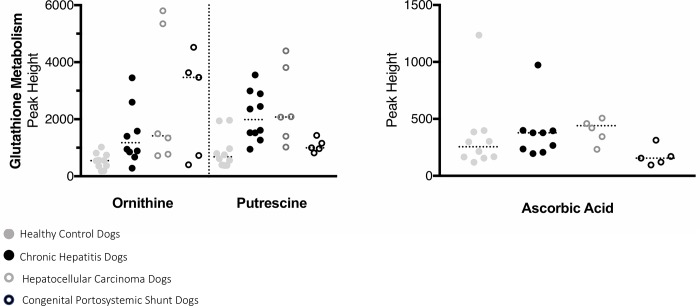
Urine metabolites of glutathione metabolism with potential value as a biomarker of chronic hepatitis, hepatocellular carcinoma, or a congenital portosystemic shunt in dogs. Each metabolite was identified by random forest and/or univariate analysis as important for the differentiation of dogs into either control, chronic hepatitis, hepatocellular carcinoma, or congenital portosystemic shunt groups. Ornithine was significantly different between groups (p = 0.013) and increased in the urine of dogs with hepatocellular carcinoma compared to control dogs (q = 0.04). Putrescine was significantly different between groups (p = 0.002) and increased in the urine of dogs with chronic hepatitis and hepatocellular carcinoma compared to control dogs (q = 0.006, q = 0.01, respectively). Ascorbic acid was significantly different between groups (p = 0.14) and increased in the urine of dogs with hepatocellular carcinoma compared to dogs with a congenital portosystemic shunt (q = 0.02).

**Fig 5:**
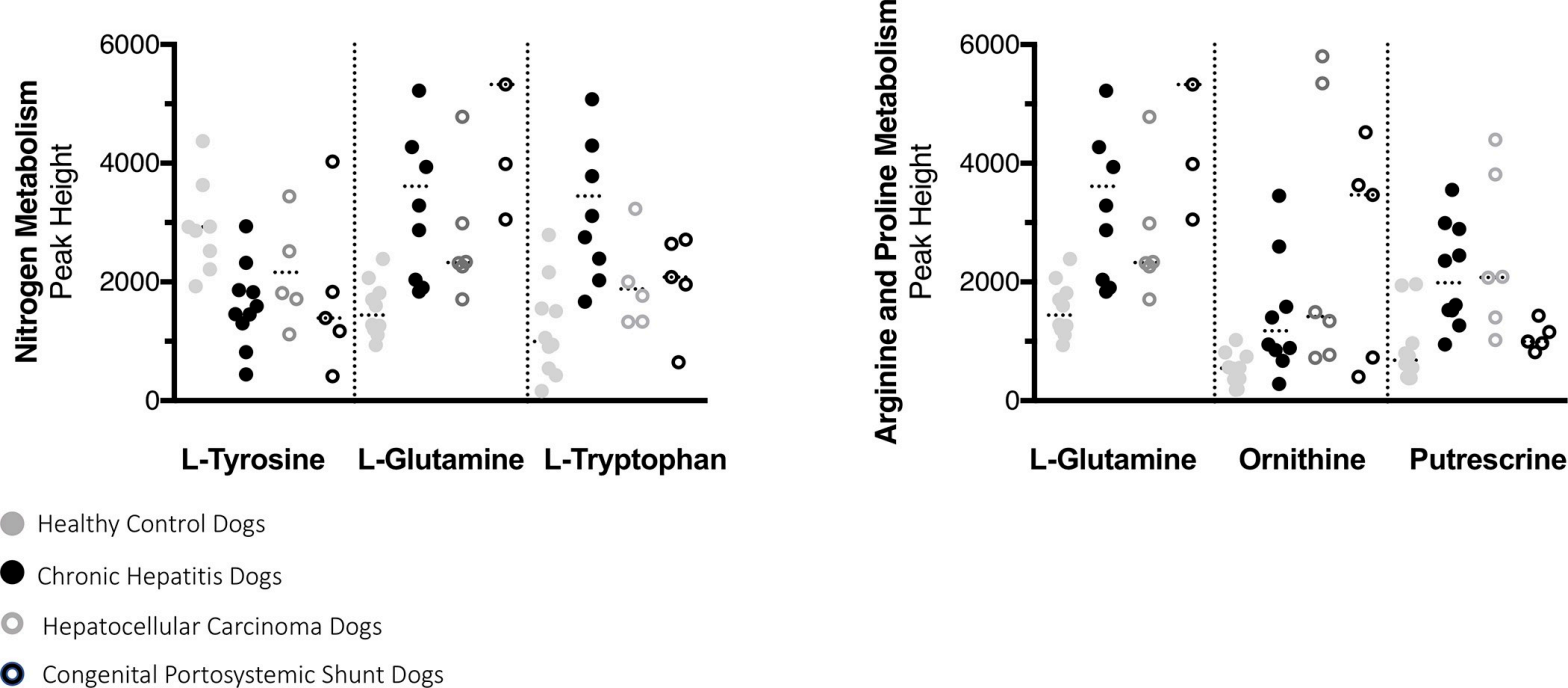
Urine metabolites of nitrogen metabolism, arginine, and proline biosynthesis with potential value as a biomarker of chronic hepatitis, hepatocellular carcinoma, or a congenital portosystemic shunt in dogs. Each metabolite was identified by random forest and/or univariate analysis as important for the differentiation of dogs into either control, chronic hepatitis, hepatocellular carcinoma, or congenital portosystemic shunt groups. Tyrosine was significantly different between groups (p = 0.01) and decreased in the urine of dogs with chronic hepatitis compared to control dogs (q = 0.01). Glutamine was significantly different between groups (p = 0.0004) and increased in the urine of dogs with chronic hepatitis and a congenital portosystemic shunt compared to control dogs (q = 0.005, q = 0.0009, respectively). Tryptophan was significantly different between groups (p = 0.004) and increased in the urine of dogs with chronic hepatitis compared to control dogs (q = 0.002).

**Fig 6:**
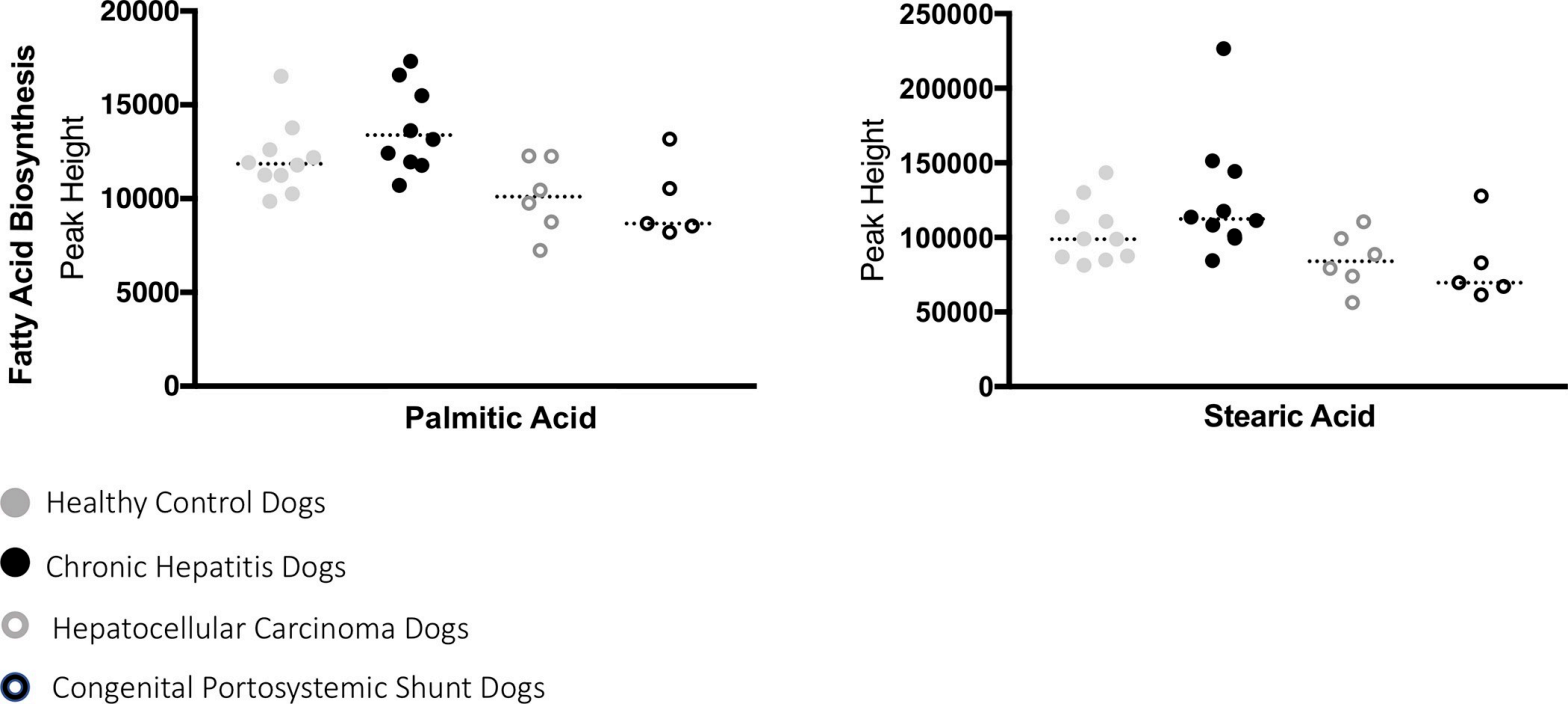
Urine metabolites of fatty acid biosynthesis with potential value as a biomarker of chronic hepatitis, hepatocellular carcinoma, or a congenital portosystemic shunt in dogs. Each metabolite was identified by univariate analysis as important for the differentiation of dogs into either control, chronic hepatitis, hepatocellular carcinoma, or congenital portosystemic shunt groups. Palmitic acid was significantly different between groups (p = 0.01) and increased in the urine of dogs with chronic hepatitis compared to dogs with hepatocellular carcinoma or a congenital portosystemic shunt (q = 0.04, q = 0.03, respectively). Stearic acid was significantly different between groups (p = 0.01) and decreased in the urine of dogs with a congenital portosystemic shunt compared to dogs with chronic hepatitis (q = 0.04).

A total of 220 named urine metabolites were identified, the abundance of which 37 differed significantly (p≤ 0.05; q<0.10) between healthy control dogs, dogs with chronic hepatitis, dogs with hepatocellular carcinoma, and dogs with a congenital portosystemic shunt (Table 3). The raw data set is available from the Dryad Digital Repository: https://doi.org/10.5061/dryad.8dg7kg5. Summary statistics of data variance and distribution are included as supporting information (S1 Table). Principle component analysis plots showed a clustering of the enrolled patients based on disease classification (Fig 1). The distribution of the most significant metabolites separating the four groups was visualized via heat map (Fig 2). Random forest analysis identified metabolites that had the highest discriminatory power between the four groups (Fig 3). Urine metabolites involved in glutathione, nitrogen, arginine, and proline metabolism and fatty acid biosynthesis that differed significantly between groups were plotted in Figs 4–6.

**Table 3:** Urine metabolites that differed significantly between healthy control dogs, dogs with chronic hepatitis, dogs with hepatocellular carcinoma, and dogs with a congenital portosystemic shunt.

Compound Name	p-value_KW	q-value_KW
**gluconic acid**	0.000251	0.021188
**succinic acid**	0.000331	0.021188
**glutamine**	0.000367	0.021188
**glucose**	0.000385	0.021188
**mannose**	0.000511	0.022470
**sorbitol**	0.000812	0.028368
**glycyl proline**	0.000904	0.028368
**valine**	0.001032	0.028368
**2-deoxypentitol**	0.001229	0.028483
**gluconic acid lactone**	0.001321	0.028483
**putrescine**	0.001457	0.028483
**cellobiose**	0.001554	0.028483
**xylitol**	0.002287	0.038710
**3-ureidopropionate**	0.003685	0.057910
**tryptophan**	0.003995	0.058586
**citric acid**	0.005117	0.069541
**adenine**	0.005374	0.069541
**ethanolamine**	0.005783	0.070113
**threonine**	0.006092	0.070113
**3-hydroxybutyric acid**	0.006374	0.070113
**heptadecanoic acid**	0.007225	0.075687
**palmitic acid**	0.009100	0.079666
**glycerol**	0.009313	0.079666
**tyrosine**	0.009734	0.079666
**lysine**	0.010036	0.079666
**2,5-dihydroxypyrazine**	0.010055	0.079666
**2-picolinic acid**	0.010088	0.079666
**pyrogallol**	0.010139	0.079666
**isomaltose**	0.010669	0.080940
**2,3-dihydroxybutanoic acid**	0.011770	0.081349
**2-hydroxybutanoic acid**	0.012612	0.081349
**ornithine**	0.012808	0.081349
**alloxanoic acid**	0.013170	0.081349
**2-deoxytetronic acid**	0.013342	0.081349
**stearic acid**	0.013582	0.081349
**ascorbic acid**	0.013668	0.081349
**glucoheptulose**	0.013681	0.081349

KW–Kruskal-Wallis H Test

## Discussion

This study identified metabolic abnormalities in the urine of dogs diagnosed with chronic hepatitis, hepatocellular carcinoma, or a congenital portosystemic shunt with significant alterations in 37 of 220 named metabolites based on univariate data analysis. These metabolites are known to be involved in a variety of processes, including glutathione metabolism, nitrogen metabolism, arginine and proline metabolism, and fatty acid biosynthesis. Principle component analysis score plot allowed visualization of the data with dogs with hepatocellular carcinoma showing the clearest separation from healthy control dogs. Dogs with hepatocellular carcinoma were easily distinguished from healthy control dogs in hierarchical cluster analysis with the heatmap approach and overlapped with metabolites extracted in random forest analysis, which is based on a different algorithm. The results provide preliminary evidence for these metabolites to be used as biomarkers in disease classification.

### Abnormal amino acid metabolism and fatty acid biosynthesis

Significant differences were noted in the relative amounts of metabolites in the urine of dogs with chronic hepatitis, hepatocellular carcinoma, or a congenital portosystemic shunt compared to healthy control dogs. Among these findings was a significant difference in three metabolites derived from glutathione metabolism, ornithine, putrescine, and ascorbic acid between groups. Ornithine was significantly increased in the urine of dogs with hepatocellular carcinoma compared to healthy control dogs (q = 0.0410). Putrescine was significantly increased in the urine of dogs with chronic hepatitis and hepatocellular carcinoma compared to healthy control dogs (q = 0.0056, q = 0.010 respectively). Ascorbic acid was significantly increased in the urine of dogs with hepatocellular carcinoma compared to dogs with a congenital portosystemic shunt (q = 0.02). Glutathione (GSH, L-γglutamyl-L-cysteinyl- glycine) is a tripeptide thiol synthesized in all mammalian cells [25]. Glutathione detoxifies reactive molecules by either spontaneous conjugation or a GSH-S-transferase catalyzed reaction. The liver, kidney, and lungs are important sources of GSH; however, systemic concentrations of GSH and its substrates are predominantly hepatically derived [26]. Hepatocytes are unique, compared with other cells, because they synthesize large quantities of GSH that can be either used locally or released into the systemic circulation and bile [26]. The discovery of increased urine concentrations of three metabolites derived from glutathione metabolism in dogs with chronic hepatitis and hepatocellular carcinoma compared to healthy control dogs and dogs with a congenital portosystemic shunt may represent altered glutathione metabolism. Decreased quantities of glutathione have been found in the liver of dogs with various liver diseases including chronic hepatitis [22]. The increased utilization of the glutathione pathway resulting in decreased liver tissue concentrations may result in increased urinary concentrations of products derived from glutathione metabolism. In addition, multiple pathways associated with glutathione metabolism were also significantly altered, including arginine and proline metabolism, and glycine, serine, and threonine metabolism. These alterations in glutathione metabolism and other glutathione-relevant pathways may reflect an effort at the cellular level to replenish GSH stores. There are no previous studies of the urine metabolome in dogs with hepatic disease; however, similar findings of increased tyrosine, glutamine, ornithine, valine, threonine, and lysine have been reported in the urine of humans with Wilson’s disease [27].

Significant differences in amino acids involved in nitrogen metabolism; tyrosine, glutamine, and tryptophan and were detected. There was a significant decrease of tyrosine in the urine of dogs with chronic hepatitis compared to healthy control dogs (q = 0.01), a significant increase of glutamine in the urine of dogs with chronic hepatitis or a congenital portosystemic shunt compared to healthy control dogs (q = 0.0045 and q = 0.0009, respectively), and a significant increase of tryptophan in the urine of dogs with chronic hepatitis compared to healthy control dogs (q = 0.0016). Glutamine, ornithine, and putrescine are also reaction intermediates of arginine and proline metabolism. Most amino acids are synthesized and degraded in the liver; thus, injury to the liver can result in abnormalities in the metabolism of amino acids and the release of amino acids from hepatocytes [27,28]. There are no previous studies of the urine metabolome in dogs with hepatic disease; however, in humans with the inflammatory liver disease the urinary amino acid concentrations of lysine, valine, threonine, and tyrosine are elevated when compared to healthy controls [29]. The results of that study are similar to the finding of our study. Alterations in amino acid metabolites are thought to represent an adaptive physiological response to hepatic stress in humans with nonalcoholic steatohepatitis and our results may represent a similar response in dogs [30].

Additionally, significant differences in two metabolites of fatty acid biosynthesis; palmitic and stearic acid were detected. There was a significant decrease of palmitic acid in the urine of dogs with hepatocellular carcinoma or a congenital portosystemic shunt compared to dogs with chronic hepatitis and a significant decrease of stearic acid in the urine of dogs with hepatocellular carcinoma compared to dogs with chronic hepatitis (q = 0.038, 0.026, and 0.035 respectively). Long chain free fatty acids (FFAs) of biologic relevance can be further divided into saturated fatty acids, monounsaturated fatty acids, and polyunsaturated fatty acids based on the presence of double bonds. The most abundant and well characterized FFAs in the context of apoptosis are palmitic acid, a 16 carbon length saturated fatty acid (C16:0), oleic acid, an 18 carbon length monounsaturated fatty acid (C18:1), and stearic acid, a 18 carbon length saturated fatty acid (C18:0) [31–33]. Saturated fatty acids can induce apoptosis in many different cell types and in hepatocytes, the saturated fatty acids, palmitic acid, and stearic acid lead to a concentration- and time-dependent lipoapoptosis [34]. Thus, the increase in these fatty acids could contribute to hepatocellular apoptosis. The role of FFAs in canine liver disease has not been characterized and therefore, the significance of these findings if validated in a targeted prospective study is unknown.

### Potential biomarkers for dogs with chronic hepatitis, hepatocellular carcinoma, or a congenital portosystemic shunt

A subset of compounds in urine were identified for their value in differentiating healthy control dogs from those diagnosed with chronic hepatitis, hepatocellular carcinoma, or a congenital portosystemic shunt. Random forest analysis identified 15 metabolites that were able to classify healthy control dogs (100% accuracy), dogs with chronic hepatitis (70% accuracy), dogs with hepatocellular carcinoma (33% accuracy), and dogs with a congenital portosystemic shunt (40% accuracy) (Fig 3). The non-invasive diagnosis of dogs with a congenital portosystemic shunt is not challenging with a sensitivity and specificity of 100% reported for the ammonia tolerance test and serum fasting ammonia concentration, respectively [35]. The potential for a subset of the metabolites identified in this study to identify dogs with chronic hepatitis or dogs with acquired portosystemic shunting prior to gross phenotypic changes should be investigated. Further evaluation of these findings in a prospective targeted study is warranted.

### Comparative research in humans with chronic hepatic disease

There are no veterinary studies that have examined the urine metabolome of dogs with chronic hepatitis; however, the urinary metabolites reported in this study represent several different pathways that have been previously identified as altered in human studies. A study of the urine metabolome in humans with hepatocellular carcinoma found changes similar to our study with L-tyrosine, L-threonine being present in higher levels and xylitol present at lower levels in the urine of patients with hepatocellular carcinoma relative to healthy controls [36]. Another study of the urine metabolome in humans with hepatocellular carcinoma phenotypically characterized the metabolome and identified as in our study changes in fatty acid biosynthesis (e.g. palmitic acid), and nitrogen metabolism (e.g. glutamine) [37]. Additionally, a study of the urine and serum metabolome of humans with chronic hepatic disease found differential expression of stearic acid, palmitic acid, tryptophan, and tyrosine between humans with chronic hepatitis or hepatocellular carcinoma compared to healthy controls similar to our study [38]. Furthermore, a metabolomic analysis of urine samples from children after Acetaminophen intoxication found multiple pathways associated with glutathione metabolism were also significantly altered, including arginine and proline metabolism, and glycine, and threonine metabolism similar to the findings in our study [39]. The consistent description of findings similar to those described in this study to findings in humans with hepatic disease suggests changes in related metabolic providing a degree of cross-validation. These promising results are hypothesis generating and require validation in a targeted study before clinical utility can be determined.

Limitations of this study include the influence of concurrent drug administration or diet on our findings; however, a study investigating the effects of dietary protein concentration on plasma and cerebrospinal fluid amino acids in dogs with portocaval shunting found that neither plasma nor cerebrospinal fluid amino acid levels were affected by changes in dietary protein [40]. Furthermore, special stains and submission of a liver biopsy specimen for copper quantification was at the discretion of the attending clinician and pathologist. Therefore, patients with copper-associated chronic hepatitis could have been missed. The results of this untargeted study will need to be validated in a prospective targeted study in dogs with chronic hepatitis, hepatocellular carcinoma, or a congenital portosystemic shunt. The metabolites in urine can be affected by the cumulative effect of complex physiological processes across all tissues in the body, it is not possible to definitively determine the organ, cell type, or intracellular compartment from which identified compounds originated. Furthermore, the impact of renal excretion versus reabsorption, and intestinal microbial metabolism on the types and quantity of compounds detected in the urine is unknown. Metabolomic investigation of the serum of dogs with chronic hepatitis, hepatocellular carcinoma, or a congenital portosystemic shunt compared to healthy control dogs would provide considerable additional insight into the metabolic abnormalities identified in this study and is currently underway.

Untargeted metabolomic profiling of urine from dogs with chronic hepatitis, hepatocellular carcinoma, and a congenital portosystemic shunt demonstrated significant semi-quantitative differences in 37 of 220 named metabolites, including those involved in glutathione, nitrogen, arginine and proline metabolism, and fatty acid biosynthesis. Further analytical validation of these results in a targeted study and determination of their utility as clinical biomarkers is warranted.

## Supporting information

S1 Table Summary statistics. (XLSX)
